# Autoinflammatory Diseases and COVID-19 Vaccination: Analysis of SARS-CoV-2 Anti-S-RBD IgG Levels in a Cohort of Patients Receiving IL-1 Inhibitors

**DOI:** 10.3390/jcm12144741

**Published:** 2023-07-18

**Authors:** Sara Bindoli, Chiara Baggio, Paola Galozzi, Filippo Vesentini, Andrea Doria, Chiara Cosma, Andrea Padoan, Paolo Sfriso

**Affiliations:** 1 Rheumatology Unit, Department of Medicine, University of Padova, 35128 Padova, Italy; sara.bindoli@phd.unipd.it (S.B.); chiara.baggio@unipd.it (C.B.); filippo.vesentini@studenti.unipd.it (F.V.); adoria@unipd.it (A.D.); 2 Laboratory Medicine Unit, Department of Medicine, University of Padova, 35128 Padova, Italy; paola.galozzi@unipd.it (P.G.); chiara.cosma@aopd.veneto.it (C.C.); andrea.padoan@unipd.it (A.P.)

**Keywords:** COVID-19, SARS-CoV-2 vaccination, anti-IL-1 drugs, anakinra, canakinumab, antibody response

## Abstract

The purpose of the study was to evaluate the antibody response after COVID-19 vaccination in patients affected by systemic autoinflammatory diseases (SAID) undertaking IL-1 inhibitors (IL-1i) compared to healthy vaccinated controls (HC). The course of COVID-19 in vaccinated patients on IL-1i was also assessed. The serological response was evaluated in SAID patients using the CLIA MAGLUMI TM 2000 Plus test after the first vaccination cycle and the booster dose. Fifty-four fully vaccinated healthcare workers were enrolled as HCs. GraphPad Prism 8 software was used for statistical analysis. All patients developed an adequate antibody response. No differences were observed between the antibody titers of patients on IL-1i and those not on IL-1i, either after the first vaccination cycle or the booster dose (*p* = 0.99), and to HC (*p* = 0.99). With increasing age, a decrease in antibody production was assessed after the second vaccine in SAID (r = 0.67, *p* = 0.0003). In general, 11.6% of SAID patients had COVID-19 after receiving vaccination. None of them developed severe disease or experienced flares of their autoinflammatory disease. In conclusion, patients receiving IL-1i develop an antibody response comparable to HC. No side effects after vaccination were observed; IL-1i was continued before and after injections to avoid flare-ups.

## 1. Introduction

The introduction of anti-SARS-CoV2 vaccination, which started in early 2021, has dramatically changed the severity course of COVID-19, not only in the general population but also in patients affected by different pathologies, such as cardiovascular diseases, diabetes, oncological disease, and rheumatological diseases (RD). In this field, a great deal of attention was directed toward those affected by autoimmune diseases, in particular those receiving conventional synthetic disease-modifying drugs (csDMARDs), biologics/target synthetic (b/ts) DMARDs, or anti-CD-20 drugs (rituximab). It is well known that patients affected by autoimmune and autoinflammatory syndromes [1] who undertake immunosuppressant and immunomodulatory therapies may have reduced immunogenicity after vaccination [2]. Indeed, those treated with mycophenolate mofetil, rituximab, abatacept, or glucocorticoids may present with reduced serological response [2]. A recent meta-analysis of 20 seroprevalence studies on SARS-CoV-2 vaccination reported the highest seroconversion rates with hydroxychloroquine and sulfasalazine. At the same time, methotrexate (either used in monotherapy or combination with other treatments) and rituximab were associated with the lowest response rates (81.9% and 36.3%, respectively); however anti-cytokine treatments were associated with a good seroconversion rate (>89%) either for TNF-alpha inhibitors, IL-6 inhibitors, IL-17 inhibitors, IL-1 receptor antagonist (anakinra) and Janus Kinases Inhibitors (JAK-I) [3]. In systemic autoinflammatory diseases (SAID), the effect of vaccination has hardly been reported. In a recent prospective study, it was observed that patients affected by severe COVID-19 and treated with anakinra alone or in combination with tocilizumab subsequently did not have an altered antibody response [4]; another study investigating the effect of vaccination on autoinflammatory diseases showed that the anti-SARS-CoV-2 vaccine was well tolerated in patients with pathologies mediated by IL-1, IL-18, and interferon (IFN)-γ, with no disease relapses requiring hospitalization, and presenting an adequate antibody production after the booster dose [5]. Regarding safety and adverse events (AEs), a recent study on Familial Mediterranean Fever (FMF) observed that 93 out of 161 patients reported adverse events/fever attacks after vaccination, with 54.7% of AEs occurring after mRNA vaccines [6]. A cross-sectional observational study in Turkey involving patients affected by FMF, Behçet Disease (BD), and rheumatic diseases (RD) other than FMF and BD observed a similar frequency of AEs in FMF/BD compared to RD. However, data on immunogenicity were not reported [7]. In general, anti-SARS-CoV-2 vaccination was well tolerated in patients with RD, with the vast majority obtaining a consistent serological response and reassuring patients treated with immunomodulatory therapies on the immunogenicity and short-term safety of the injections [8]. The present work aims to evaluate the levels of SARS-CoV-2 Spike protein Receptor Binding Domain (S-RBD) IgG antibody in a group of patients treated with IL-1 inhibitors (IL-1i), anakinra and canakinumab to observe whether IL-1i may be associated or not with a reduced serological response compared to a group of fully vaccinated healthy controls (HCs).

## 2. Materials and Methods

### 2.1. Clinical and Demographic Characteristics of the Patients Included in the Study

**Figure 1:**
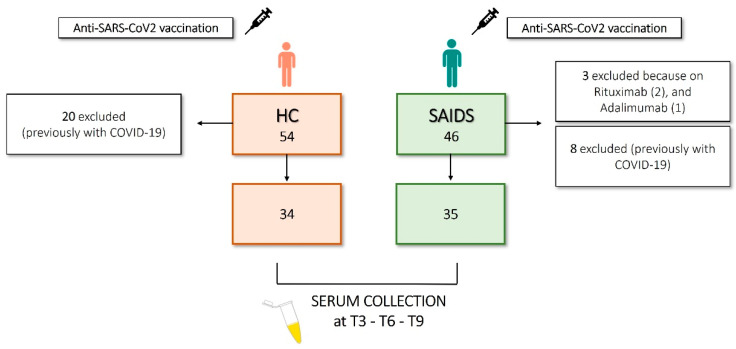
Flow diagram illustrating study size and participants.

Forty-six SAID patients and fifty-four age- and sex-matched HCs were included in the study. The demographical and clinical data of SAID patients are depicted in Table 1 and graphically presented in Figure 1. SAID patients were included if they were injected with the anti-SARS-CoV-2 vaccine and if they were treated with IL-1i (anakinra or canakinumab) alone or in combination with colchicine or glucocorticoids (GCs) if they received colchicine, GCs, or if they were not on treatment for disease remission (but previously treated with colchicine or GCs). No relevant comorbidities (cardiovascular or oncological pathologies, pre-existing lung disease, or diabetes) were reported in the cohort, and disease activity remained stable throughout the study. Three patients were excluded from the study because they did not receive IL-1i or colchicine but were on therapy with rituximab (two patients) and adalimumab. Eight out of 43 (18.6%) patients had a previous diagnosis of COVID-19 infection (COVID+), based on the positivity to nasopharyngeal swab test, and therefore were excluded from the analysis. Concerning the type of vaccine, 40 patients received Comirnaty BNT162b2 mRNA (BioNTech-Pfizer, Mainz, Germany/New York, NY, USA), 2 received mRNA Spikevax 1273 (Moderna, Cambridge, MA, USA), and one patient received AZD1222 ChAdOx1 (University of Oxford/AstraZeneca, Oxford/Cambridge, UK) at the first two doses. HCs were chosen among healthcare workers of Padova University Hospital and were not affected by relevant comorbidities. The mean age was 40 (range, 25–67) years with a standard deviation (SD) of ±11 years. All of them received the Comirnaty BNT162b2 mRNA vaccine. Among the HCs included in this study, 33 (61%) were women, and 21 (39%) were men. A total of 20/54 (37%) HCs had a previous diagnosis of COVID-19 infection (COVID+), based on the positivity to nasopharyngeal swab test and therefore excluded from the analysis. Patients and HCs underwent a primary vaccination cycle at the local vaccination hub; for SAID patients, the first dose was followed by a second after 21 days, between March and May 2021; the booster dose was between October and December 2021. For HCs: first dose, followed by a second after 21 days, between January and March 2021; booster dose between October and December 2021. Blood samples were prospectively collected in June 2021 (T3), October–November 2021 (T6), and February–April 2022 (T9). The biological samples obtained from patients and HCs were deidentified. Patients’ sera were collected at Rheumatology Unit and sent to Laboratory Medicine Unit for analysis. The anti-SARS-CoV-2 Spike protein receptor binding domain (S-RBD) IgG levels were assessed in SAID and HCs at different time points (T3, T6, and T9). For this reason, we decided to conduct the subsequent analysis only in COVID-negative SAID and HCs. All the subjects gave their fully informed written consent to participate in the study, which was carried out per the Declaration of Helsinki. The protocol follows the guidelines of the Ethics Committee of Padova University Hospital. The flow diagram depicted in Figure 1 shows the study size and the number of participants included in the final analysis.

### 2.2. Evaluation of Binding IgG Antibodies against the RBD Portion of the SARS-CoV-2 Spike Protein

SARS-CoV-2 S-RBD IgG were measured by chemiluminescent immunoassays (CLIA) on Maglumi 2000 plus (Snibe Diagnostics, Shenzhen, China), validated elsewhere [9], with results expressed in kilo Binding Antibody Unit (kBAU). The cutoff value is 33.0 kBAU/mL. Thus values ≥ 33.0 kBAU were accepted as positive and <33.0 kBAU as negative. IgG levels in SAID patients were measured 2–4 weeks after the second dose (T3), six months after the second dose (T6), and 90 days after the third dose (T9). Due to technical issues (limited reagent availability) and reduced compliance of some SAID patients, not all samples were assessed at each time point. Furthermore, not all SAID patients completed the analysis due to the limited ability to reach the Hospital during the pandemic. Therefore, the number of samples evaluated for SAID patients was: 43/43 (100%) in T3, 28/43 (65%) in T6, and 23/43 (54%) in T9. The numbers of samples evaluated for HCs were 48/54 (89%) in T3, 54/54 (100%) in T6, and 54/54 (100%) in T9.

### 2.3. Statistical Analysis

Data are reported as the median and interquartile range (IQR). The Shapiro-Wilk test was used to analyze the distribution of continuous variables. As data distribution was non-normal, Kruskal Wallis followed by Dunnet post hoc tests were used for multiple comparisons. Spearman correlation analysis was used to determine the correlations. Statistical analysis was performed with GraphPad Prism 8 (GraphPad Software Inc., La Jolla, CA, USA). A *p*-value < 0.05 was considered significant.

## 3. Results

**Figure 2:**
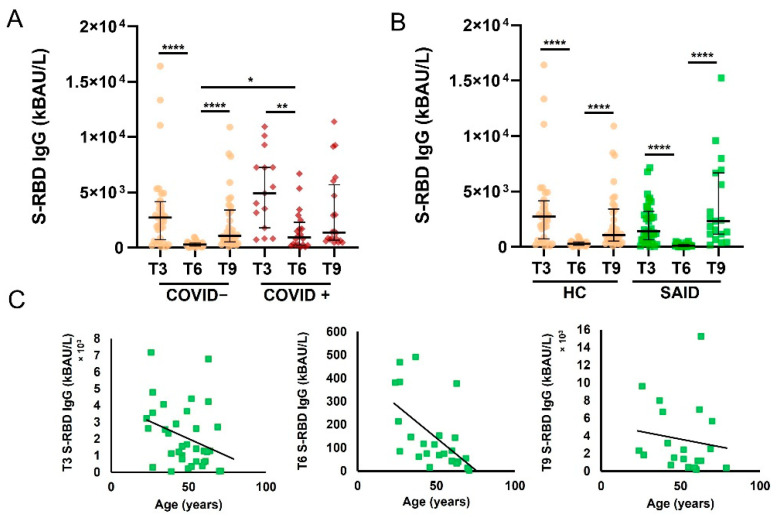
Levels of S-RBD IgG at different time points in patients with SAID and HC. (**A**). S-RBD IgG levels in COVID and COVID+ HC were measured at T3, T6, and T9. Data are reported as median and IQR. *p* was calculated according to the Kruskal-Wallis test. Dunn post hoc test, **** *p* < 0.0001, ** *p* < 0.01, * *p* < 0.05. (**B**). S-RBD IgG levels in COVID− SAID patients and COVID-HCs were measured at T3, T6, and T9. Data are reported as median and IQR. *p* was calculated according to the Kruskal-Wallis test. Dunn’s post hoc test, **** *p* < 0.0001. (**C**). Correlation of S-RBD IgG levels in SAID patients measured at T3, T6, T9, and age. Spearman correlation analysis was used to determine the correlations. HC, healthy controls; SAID, Systemic autoinflammatory diseases; IQR, interquartile range.

We evaluated the anti-SARS-CoV-2 Spike protein receptor binding domain (S-RBD) IgG levels in SAID patients and HCs at different time points (T3, T6, and T9). As previously reported by Padoan et al. [10], S-RBD IgG levels were significantly higher in COVID+ HCs than in COVID− HCs (*p* < 0.05) (Figure 2A). For this reason, we decided to perform the subsequent analysis only in COVID-negative SAID and HCs. We evaluated S-RBD IgG levels in patients with SAID and HCs at different time points (Figure 2B). The number of samples evaluated for SAID patients was: 35/35 (100%) in T3, 24/35 (69%) in T6, and 19/35 (54%) in T9. In both analyzed groups (HC and SAID patients), the S-RBD IgG levels were significantly higher at T3 and T9 than at T6 (*p* < 0.0001). We found no differences between the patient group and the HCs at any of the time points considered. No significant associations were found between age and S-RBD IgG levels in T9 (*p* = 0.61) in SAID patients, while a negative correlation was found between age and S-RBD IgG in T6 (*p* = 0.0003, r = −0.67) (Figure 2C). Median levels of S-RBD IgG and interquartile range (IQR) of HCs and SAID patients are shown in Table 2.

**Figure 3:**
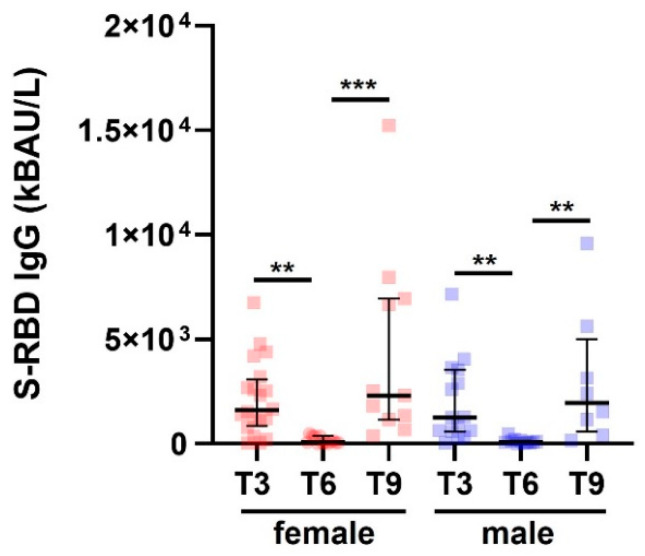
S-RBD IgG levels in patients with SAID and subdivided by sex. S-RBD IgG levels in SAID patients (COVID−) divided by gender measured at T3, T6, and T9. Data are reported as median and IQR. *p* was calculated according to the Kruskal-Wallis test. Dunn post hoc test, ** *p* < 0.01, *** *p* < 0.0003. SAID, Systemic autoinflammatory diseases. IQR, interquartile range.

Among the COVID- SAID patients included in this study, 57% were females, and 43% were males. The overall mean value for age, which did not differ significantly by sex (Mann-Whitney, *p* = 0.67), was 48 years with a standard deviation (SD) of ±14 years. The number of samples evaluated for female SAID patients was: 20/20 (100%) in T3, 13/20 (65%) in T6, and 11/35 (31%) in T9. The number of samples evaluated for male SAID patients was: 15/15 (100%) in T3, 11/15 (65%) in T6, and 8/15 (31%) in T9. Table 2 and Figure 3 show that S-RBD IgG levels did not differ between male and female patients with SAID.

**Figure 4:**
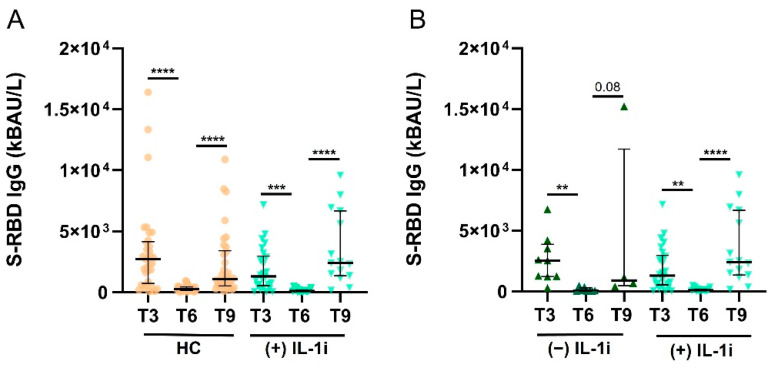
Levels of S-RBD IgG in SAID patients treated with IL-1i. (**A**). S-RBD IgG levels were measured in COVID− HC and COVID− SAID patients treated with IL-1i at T3, T6, and T9. Data are reported as median and IQR. *p* was calculated according to Kruskal-Wallis’s test. Dunn post hoc test, **** *p* < 0.0001, *** *p* < 0.001. (**B**). S-RBD IgG levels in SAID patients treated or not with IL-1i were measured at T3, T6, and T9. Data are reported as mean ± SEM. *p* was calculated according to Kruskal-Wallis’s test. Dunn post hoc test, ** *p* < 0.005, **** *p* < 0.0001. HC, healthy controls; SAID, Systemic autoinflammatory diseases; IL-1i, interleukin 1 inhibitors, IQR, interquartile range.

Finally, we evaluated S-RBD IgG levels in COVID-negative patients treated with IL-1i. Figure 4A shows no significant differences between S-RBD IgG levels in T3, T6, and T9 in SAID patients treated with IL-1i and HCs. Furthermore, no significant differences were observed in S-RBD IgG levels in SAID patients treated with IL-1i and those who did not receive these treatments (Figure 4B). The number of samples evaluated for SAID treated with IL-1i was: 26/26 (100%) at T3, 16/26 (62%) at T6, and 15/26 (58%) at T9. The number of samples evaluated for patients with SAID not treated with IL-1i was: 9/9 (100%) in T3, 8/9 (89%) in T6, and 4/9 (44%) in T9.

Through e-mail or telephone surveillance, patients were asked to report if they had disease relapses after anti-SARS-CoV-2 injection; none of them complained of hyperpyrexia, joint manifestations, skin rash, or serositis after vaccination, in addition to canonical injection site reactions as observed in the general population, or mild fever and arm pain for a few days after the injection. Regarding the five patients with a breakthrough infection, no disease relapses were observed during COVID-19, and none discontinued IL-1i. Table 3 illustrates the clinical features of COVID-19 in the five SAID infected and the ongoing therapies.

## 4. Discussion

The introduction of SARS-CoV-2 vaccination has profoundly changed the clinical outcome of those affected. It aims to prevent the more severe consequences of the infection itself, either in the general population or in patients affected by RD. Patients receiving immunomodulatory treatments are usually considered to have an increased risk of infections, and several factors such as older age, male sex, comorbidities, and the intake of a high dose of glucocorticoids (e.g., prednisone > 10 mg/day) or B-cell depleting therapies may increase the risk of hospitalization and death [11,12]. However, several biologics used in cohorts of patients with RD have been associated with a lower risk of hospitalization. Furthermore, different immunomodulatory therapies have been evaluated in the last two years as potential strategies for hyperinflammation, also called the ‘cytokine storm’, caused by SARS-CoV2, and have shown beneficial effects to date [13]. With the results obtained from our analysis, it is possible to observe that patients receiving anakinra and canakinumab did not show reduced antibody production after vaccination after both the complete cycle (I-II dose) and the booster dose, with titers that are compatible with those observed in HCs. A significant reduction in antibody levels was reported at T6 (6 months after the second injection) compared to T3, but this result is consistent with the normal decrease in serological response commonly observed in the general population and, in our case, in HCs. In T9, SAID patients presented with higher antibody titers. However, the difference was not significant compared to HCs. This data may be interpreted as a bias since SAID patients analysed in T9 were fewer than in T3, and the variability was elevated. Regarding the age differences between SAID patients and controls, we observed a reduction in T6 titer in both groups, in line with the increase in age. Overall, in this cohort, no patient was a no responder, and all 43 subjects developed antibody titers that exceeded the established cutoff, fixed at 33 kBAU/L, and were adequate to protect the patients from serious infection. Our data are consistent with other reports in which the serological response to vaccination was not impaired in those treated with IL-1i [14]. Similar results were also observed in adults treated with canakinumab who received influenza and meningococcal vaccines and had antibody titers comparable to controls [15]. Furthermore, comprehensive data on the safety and efficacy of inactivated vaccines were also reported in children taking IL-1i [16,17]. Therefore, patients on therapy with IL-1i should be encouraged not to discontinue the treatment during SARS-CoV-2 vaccination [18], given the risk of disease relapses and the possible additional increase of IL-1 if breakthrough infections by COVID-19 appear. Regarding safety and AEs, in a real-life observational study reported by Peet et al. and carried out in autoinflammatory patients treated with different biologics (IL-1i, IL-6 inhibitors, TNF-alpha inhibitors), side effects after vaccination against COVID-19 were reported after 71 of 138 (51.4%) administrations and were consistent with a flare of the underlying disease only in 26 of 138 (18.8%). Fatigue, myalgia, headache, and fever were the most frequent side effects observed after mRNA and adenovirus vaccines, but no serious AEs or death were reported [19]. Similarly, in the cohort reported by Shechtman et al., 273 FMF patients vaccinated with the BNT162b2 vaccine reported local reactions and mild systemic events; moreover, the Authors observed a recurrence of FMF-attacks (mostly abdominal serositis and joint involvement) after two doses in 12% of the subjects. However, the disease activity remained stable in most patients, with a rate of attacks higher the month before the vaccination than that after vaccination [20]. Moreover, they also observed that FMF patients treated with colchicine and canakinumab had a significantly higher rate of attacks following vaccination than those treated with colchicine alone. The explanation given is that patients under combination therapy usually have a high disease activity, thus, are more prone to develop attacks, but it is not an effect of IL-1 inhibition itself [20]. Güven and Colleagues observed a considerable number of FMF patients (54.7%) who suffered from vaccine-related adverse events and/or FMF attacks (mostly abdominal pain and fever), especially after receiving BNT162b2. They observed an increased rate of AEs after the booster dose. However, no serious events or increased mortality due to vaccination were detected [6]. Episodes of serositis (peritonitis, pleuritis), fever attacks and articular manifestations were observed in the Turkish cohort of 247 FMF patients reported by Ozdede et al.: an overall rate of 13.4% flares occurred after the BioNTech vaccine; however, in general, a similar AEs profile and frequency was observed in FMF/BD patients when compared to RD patients [7]. Concerning COVID-19 breakthrough infections, 5 out of 43 patients (11.6%) were infected with SARS-CoV-2 after the booster dose (third dose) but did not exhibit severe manifestations of COVID-19 nor disease flare-ups or symptoms related to long COVID-19 in the follow-up (Table 3). This could be explained by the fact that they received three vaccine shots. Therefore, the course of the disease resulted in mild; however, they all received IL-1i too, which could have down-regulated an eventual hyperinflammatory response caused by SARS-CoV-2. It is well-assessed that IL-1i, especially anakinra, may be beneficial in dampening the cytokine boost typical of the severe phases of COVID-19 infection. Therefore, the already in-place blockage of the inflammasome-IL-1β axis and the effect exerted by vaccination could have protected these patients more from developing a major infection and, in turn, from hyperinflammation. Our data are consistent with the clinical profile of SARS-CoV-2 breakthrough infections in double or triple-vaccinated, in which a lower rate of hospitalization and fatal outcome was reported compared to unvaccinated RD patients [21]. However, the sample size is too small to conclude the positive effect exerted by IL-1i, but the intake of certain drugs should be considered. Notably, in this cohort, two patients affected by IgG4-related diseases and receiving rituximab were not included in the analysis, as it is widely recognized that anti-CD-20 drugs can severely hamper the serological response [22]. No differences were observed between patients on IL-1i and those on colchicine or not treated. Almost all patients (42/43, 97.6%) received mRNA vaccines, and only one subject received the adenovirus vectored vaccine (AZD1222) in the first cycle but received a dose of mRNA vaccine at the booster dose (heterologous vaccination). Regarding safety, none of our patients treated with IL-1i or colchicine developed disease flares or serious AEs after receiving anti-SARS-CoV-2 vaccination, data consistent with the previously discussed literature. According to the EULAR recommendation for vaccination in subjects with RD, we suggested the administration of the vaccine to patients with ‘quiet disease’ (low disease activity or clinical remission), and we advise not discontinuing medications during the vaccination period to avoid possible disease flare-ups [23].

## 5. Conclusions

In conclusion, the present study supports that the anti-SARS-CoV-2 vaccines are equally immunogenic in SAID and HCs. Treatment with IL-1i is not associated with a delayed or hampered serological response, and no adverse events were observed after vaccination in those treated, providing evidence of excellent safety and tolerance in patients affected by autoinflammatory diseases. A limitation of the study is the small sample size of patients enrolled. However, considering that SAIDs are rare pathologies, the total number of patients is acceptable to present sufficient data on the vaccine’s tolerability and efficacy during IL-1i. Further studies are necessary to understand if a fourth and a fifth dose or even annual vaccination against SARS-CoV-2 will be necessary for patients with RD or if guidelines for the general population should be followed.

## Figures and Tables

**Table 1:** Characteristics of SAID patients included in the study.

**Demographic Characteristics**	
Patients, *n*	43
Age, years (IQR)	49 (37–60)
Sex, *n* (%)	F, 26 (60.5); M, 17 (39.5)
**Type of SAID**	
AOSD, *n* (%)	20 (46.5)
FMF, *n* (%)	17 (39.5)
TRAPS, *n* (%)	3 (7.0)
CAPS, *n* (%)	2 (4.7)
Schnitzler, *n* (%)	1 (2.3)
**Therapy**	
IL-1i (alone), *n* (%)	18 (42.8)
IL-1i + colchicine, *n* (%)	6 (14)
IL-1i + GCs < 10 mg/day, *n* (%)	4 (9)
IL-1i + JAK inhibitors, *n* (%)	1 (2)
IL-1i + methotrexate, *n* (%)	2 (4)
Colchicine, *n* (%)	6 (14)
GCs < 10 mg/day, *n* (%)	1 (2)
None, *n* (%)	5 (11)
**Type of Vaccine**	
mRNA Comirnaty BNT162b2 (%)	40 (93.0)
mRNA Spikevax 1273 (%)	2 (4.7)
AZD1222 ChAdOx1 (%)	1 (2.3)

Data are expressed as the median and interquartile range (IQR) or number of patients (*n*) and percentage (%). IL-1i, IL-1 inhibitors; AOSD, Adult-Onset Still’s Disease; FMF, Familial Mediterranean Fever; GCs = glucocorticoids; TRAPS, TNF receptor-associated periodic syndrome; CAPS, Cryopyrin-Associated Autoinflammatory Syndromes; IQR, interquartile range; *n*, number of patients.

**Table 2:** Anti-SARS-CoV-2 spike protein receptor binding domain (S-RBD) IgG levels at different time points in SAID patients and HC.

		T3	T6	T9
HC	S-RBD IgG, kBAU/L (IQR)	2719 (724–4154) *	275 (149–443)	1077 (529–3409) *
female		2934 (1690–4796) *	345 (137–514)	1150 (529–3755) °
male		1878 (413–3334) #	197 (147–296)	828 (497–2943) #
SAID, patients	S-RBD IgG, kBAU/L (IQR)	1400 (637–3206) *	86 (45–198)	2316 (1155–6681) *
female		1600 (852–3079) #	74 (43–378)	2316 (1155–6938) °
male		1261 (595–3548) #	87 (42–152)	1969 (594–5005) #

S-RBD IgG levels in COVID− SAID patients and COVID− HC were measured at T3, T6, and T9 using CLIA Maglumi 2000 plus as described in Materials and Methods. Data are reported as median and IQR. *p* was calculated according to the Kruskal-Wallis test. Dunn post hoc test, * *p* < 0.0001, ° *p* < 0.001, # *p* < 0.01 vs. T6. HC, healthy controls; SAID, Systemic autoinflammatory diseases; IQR, interquartile range.

**Table 3:** The course of COVID-19 in 5 SAID patients after receiving three injections of anti-SARS-CoV-2 vaccine.

Patients	1	2	3	4	5
Disease	TRAPS	FMF	AOSD	FMF	AOSD
Date of the booster dose	21 November	21 November	21 December	21 November	21 December
Date of infection	22 January	22 May	22 January	22 March	22 June
COVID-19 symptoms	Sore throat, running nose	Fever < 38°, sore throat, cough, running nose	Fever < 38°, cough, running nose	Arthralgias, sore throat	Fever < 38°, sore throat
Therapy during COVID-19	CAM 300 mg/4 weeks	CAM 300 mg/4 weeks	Anakinra 200 mg/day	CAM 150 mg/4 weeks	Anakinra 100 mg/day
Therapy for COVID-19	Antipyretics	Antipyretics	Antipyretics	Monoclonal antibodies	Antipyretics

TRAPS, TNF-associated periodic syndrome; FMF, familial Mediterranean fever; AOSD, adult-onset Still’s disease; CAM, canakinumab.

## Data Availability

Not applicable.
